# Astragalus polysaccharides attenuate rat aortic endothelial senescence via regulation of the SIRT-1/p53 signaling pathway

**DOI:** 10.1186/s12906-024-04387-4

**Published:** 2024-02-08

**Authors:** Xinyu Miao, Lingjun Rong, Bo Fu, Shaoyuan Cui, Zhaoyan Gu, Fan Hu, Yanhui Lu, Shuangtong Yan, Banruo Sun, Wenli Jiang, Yuting Zhang, Yanping Gong, Chunlin Li

**Affiliations:** 1 Department of Endocrinology, The Second Medical Center & National Clinical Research Center for Geriatric Diseases, Chinese PLA General Hospital, Beijing, P.R. China; 2 Department of Nephrology, The First Medical Center, State Key Laboratory of Kidney Diseases, Chinese PLA General Hospital & Chinese PLA Institute of Nephrology, National Clinical Research Center for Kidney Diseases, Beijing, P.R. China; 3 School of Life Sciences, Hebei University, Baoding, Hebei P.R. China

**Keywords:** Astragalus polysaccharides, Endothelial cell, Senescence, Oxidative stress, SIRT-1

## Abstract

**Background:**

Astragalus polysaccharides (APS) have been verified to have antioxidative and antiaging activities in the mouse liver and brain. However, the effect of APS on aortic endothelial senescence in old rats and its underlying mechanism are currently unclear. Here, we aimed to elucidate the effects of APS on rat aortic endothelial oxidative stress and senescence in vitro and in vivo and investigate the potential molecular targets.

**Methods:**

Twenty-month-old natural aging male rats were treated with APS (200 mg/kg, 400 mg/kg, 800 mg/kg daily) for 3 months. Serum parameters were tested using corresponding assay kits. Aortic morphology was observed by staining with hematoxylin and eosin (H&E) and Verhoeff Van Gieson (VVG). Aging-related protein levels were evaluated using immunofluorescence and western blot analysis. Primary rat aortic endothelial cells (RAECs) were isolated by tissue explant method. RAEC mitochondrial function was evaluated by the mitochondrial membrane potential (MMP) measured with the fluorescent lipophilic cationic dye JC‑1. Intracellular total antioxidant capacity (T-AOC) was detected by a commercial kit. Cellular senescence was assessed using senescence-associated-β-galactosidase (SA-β-Gal) staining.

**Results:**

Treatment of APS for three months was found to lessen aortic wall thickness, renovate vascular elastic tissue, improve vascular endothelial function, and reduce oxidative stress levels in 20-month-old rats. Primary mechanism analysis showed that APS treatment enhanced Sirtuin 1 (SIRT-1) protein expression and decreased the levels of the aging marker proteins p53, p21 and p16 in rat aortic tissue. Furthermore, APS abated hydrogen peroxide (H_2_O_2_)-induced cell senescence and restored H_2_O_2_-induced impairment of the MMP and T-AOC in RAECs. Similarly, APS increased SIRT-1 and decreased p53, p21 and p16 protein levels in senescent RAECs isolated from old rats. Knockdown of SIRT-1 diminished the protective effect of APS against H_2_O_2_-induced RAEC senescence and T-AOC loss, increased the levels of the downstream proteins p53 and p21, and abolished the inhibitory effect of APS on the expression of these proteins in RAECs.

**Conclusion:**

APS may reduce rat aortic endothelial oxidative stress and senescence via the SIRT-1/p53 signaling pathway.

## Background

The prevalence of atherosclerosis and cardiovascular disease is markedly increased with advancing age. Age-related vascular endothelial dysfunction is an early pathophysiological hallmark of cardiovascular disease and contributes to disease progression and poor outcomes, such as myocardial infarction and heart failure. Oxidative stress is known to play an important role in the aging process [1–3], can result in vascular endothelial dysfunction which can be reflected by nitric oxide (NO) bioavailability adjusted by endothelial nitric oxide synthase (eNOS) [4, 5].

Astragalus polysaccharides (APS) are the major active components of Radix Astragali (Latin binomial nomenclature is Astragalus mongholicus Bunge), dried root of Astragalus membranaceus Bge. var. mongholicus Hsiao (A. mongholicus) or Astragalus membranaceus (Fisch) Bge. (Leguminosae) which has been a commonly used traditional herb for more than 2000 years in China. APS have a variety of biological activities, including antioxidative, anti-inflammatory, anti-aging and cardioprotective activities [6–8]. Moreover, APS have been proven to promote the secretion of NO and the synthesis of eNOS in rat pulmonary artery [9]; exert an anti-aging effect through antioxidative properties, such as lowering reactive oxygen species (ROS) production in bone marrow mesenchymal stem cells (BMSCs) and mitochondria in mouse liver and brain [10–12]; and prolong the silkworm lifespan by mitigating endoplasmic reticulum stress [13]. We found that APS reduce high-glucose-induced vascular endothelial cell senescence by modulating the mitochondrial Na^+^/Ca^2+^ exchanger recently [14], however, the effects of APS on aortic endothelial oxidative stress and senescence in aged rats are currently unclear.

Sirtuin 1 (SIRT-1), a NAD^+^-dependent histone deacetylase, has been linked to regulation of aging, reactions to oxidative stress, inflammation and metabolism [15, 16]. Studies have demonstrated that enhancement of SIRT-1 expression can extend lifespan in yeast, *Caenorhabditis elegans* and mice [17–19]. Moreover, SIRT-1 expression is decreased in senescent vascular tissues, and the low expression of SIRT-1 in endothelial cells accelerates mouse vascular aging [20]. APS have been reported to ameliorate oxidative stress-induced muscle mitochondrial dysfunction through the SIRT-1 pathway and attenuate ochratoxin A-induced immune stress via activation of the AMP-activated protein kinase (AMPK)/SIRT-1 signaling pathway in porcine alveolar macrophages [16, 21]. Whether SIRT-1 activation is involved in APS modulation of aortic endothelial senescence has not been elucidated. Here, we treated natural aging rats and primary aortic endothelial cells with APS to investigate the anti-aging effect of APS and its underlying mechanism.

## Methods

### Animals and treatment

Two-month-old male Wistar rats weighing 150–200 g and 9-month-old male Wistar rats weighing 300 to 400 g were obtained from Beijing Vital River Laboratory Animal Technology Co., Ltd. (Beijing, China). They were maintained in a specific-pathogen**-**free (SPF) barrier facility at atmospheric pressure with 50% relative humidity and a 12 h light and 12 h dark cycle at 22 °C. The rats received SPF rat chow and were allowed to drink sterile water ad libitum. Care providers, experimenters and data analysts were blind to the treatment. The rats were weighed and observed for general appearance during the study period. The aged rats (20 months old) were randomly divided into 4 groups: (i) control (Con) group (*n* = 6), which was treated with normal saline by gavage at a dose equal to the treatment group; (ii) APS-low dose (APS-L) group (*n* = 6), treated with a single dose of 200 mg/kg/d APS by gavage; (iii) APS-moderate dose (APS-M) group (*n* = 6), treated with a single dose of 400 mg/kg/d APS by gavage; and (iv) APS-high dose (APS-H) group (*n* = 6) treated with a single dose of 800 mg/kg/d APS by gavage. APS were obtained from Shanghai Acmec Biochemical Technology Co., Ltd. (Shanghai, China) as a white-colored and water-soluble powder, and its purity ≥ 98% with a molecular weight of 254.69. APS were authenticated by Dr. Zhenzhen Jia and also deposited in Chinese Medicine Research Institute of the Fifth Medical Center, Chinese PLA general hospital according to Chinese Pharmacopoeia (The Pharmacopoeia Commission of PRC, 2015). The APS consist of α-1,4 (1,6) dextrans, arabinogalactan (AGs), rhamnogalacturonan I (RGIs) and arabinogalactan-proteins (AGPs) compositions, and their monosaccharide compositions are mainly composed of glucose, arabinose, galactose, rhamnose and galacturonic acid, among which glucose, arabinose and galactose are the main components, accounting for more than 90% of the whole composition.

### Serum parameters assays

Rats were anesthetized by intraperitoneal injection of sodium pentobarbital (50 mg/kg). Blood samples from rats were collected in heparinized tubes from the abdominal aorta. Sera were obtained by centrifugation at 3000–4000 rpm for 10 min and stored at -80 °C. Alanine aminotransferase (ALT), creatinine (Cr), blood glucose (Glu), low-density lipoprotein cholesterol (LDL-C), NO, catalase (CAT) and peroxidation malondialdehyde (MDA) levels were quantified using corresponding assay kits (Nanjing Jiancheng Bioengineering Institute, Nanjing, China); eNOS and SIRT-1 levels were measured using enzyme-linked immunosorbent assay (ELISA) kits (Elabscience Biotechnology, Wuhan, China). All measurements were performed according to the manufacturer’s instructions.

### Histology and immunohistochemistry

Tissues were excised from the rat aortae and fixed in 4% formalin buffer. Then, paraffin-embedded blocks were prepared, and sections were cut at a microtome setting of 4 μm thickness and stained with hematoxylin and eosin (H&E) and Verhoeff Van Gieson (VVG) according to a previous routine method [22]. Aorta tissue slices were observed using a digital color video camera (Nikon) attached to an optical microscope (Olympus). Six visual fields in each section were randomly selected for observation. Vascular wall thickness was measured using ImageJ software (NIH, Bethesda, MD, USA).

### Immunofluorescence

Tissues from the rat aortae were fixed overnight in 4% PFA at 4 °C, embedded in OCT compound (Tissue-Tek) and cryosectioned for staining. Sections were air-dried for 2 h, washed 3 times in PBS, permeabilized in 0.5% Triton-X, and blocked for 15 min with 1% BSA in PBS. Fixed tissues were incubated at 4 °C overnight with primary antibodies against p21 (1:100, Abcam) and p16 (1:200, Abcam), and then incubated with secondary antibodies (Cy3-conjugated anti-rabbit IgG, 1:200, Beyotime, Beijing, China) at room temperature for 2 h. Nuclei were stained with 4’,6-diamidino-2-phenylindole (DAPI) (Abcam, Cambridge, MA). Images were captured using a fluorescence microscope (Olympus, Tokyo, Japan).

### Vasodilator activity

The freshly isolated thoracic aorta was placed into ice-cold Krebs-Henseleit solution containing 118.4 mmol/L NaCl, 4.7 mmol/L KCl, 4 mmol/L NaHCO_3_, 1.2 mmol/L MgSO_4_, 2 mmol/L CaCl_2_, 1.2 mmol/L KH_2_PO_4_, 10 mmol/L Hepes, and 6 mmol/L glucose, and then cut into 0.3-0.5 cm wide rings. The aortic rings were mounted between stainless steel hooks and suspended in 5 ml water-jacketed organ baths containing oxygenated Krebs-Henseleit solution at 37 °C. The tissues were allowed to equilibrate for 30 min at 80 mmHg. To measure the relaxation response, the samples were contracted in advance with a concentration of 10^− 6^ mol/L phenylephrine (Sigma, USA) that caused maximum contraction, and to complete a dose-response curve of acetylcholine for each aorta, increasing concentrations (10^− 8^ ~10^− 4^ mol/L) of acetylcholine were then added into the bath to determine the endothelial-dependent vasodilation. All the samples showed maximum vasodilation with a concentration of 10^− 6^ mol/L acetylcholine. Isometric tensions of the aortae were tested by a Grass FT03 force-displacement transducer. The responses caused by the samples were expressed as a percentage of the decrease in the initial maximum contraction force stimulated with phenylephrine.

### Detection of SIRT-1 activity

SIRT-1 activity in aortic tissue lysate was measured using the SIRT-1 fluorometric kit (Abcam, Cambridge, MA) according to the manufacturer’s instructions. Briefly, the assays were performed by incubating the tissue lysate and Fluoro-Substrate Peptide (2 × 10^–4^ mol/L), Developer, and NAD (2 × 10^–3^mol/L) at 37 °C for 30 min. Fluorescence intensity was continuously read for 30 min at 2- min intervals with excitation at 350 nm and emission at 460 nm using a microplate reader (BioTek, USA).

### Isolation, culture, and identification of rat aortic endothelial cells

Aortae were isolated from the thoracic cavity of the 2-month-old rats, opened longitudinally, dissected into 0.2–0.5 cm sections, and placed on a six-well plate with the intimal side down. The wells contained 50 µL of endothelial cell growth medium containing 15% fetal bovine serum and 100 U/mL penicillin-streptomycin (ScienCell, Carlsbad, CA). Rat aortic sections were incubated in a humidified atmosphere containing 5% CO_2_ at 37 °C. Cells were harvested after 72 h. RAECs were identified by platelet endothelial cell adhesion molecule-1 (CD31; Abcam, Cambridge, MA) immunofluorescence staining, as described in our previous study [23]. The procedure of isolating primary senescent RAECs from 20-month-old rats was the same as above.

#### Western blot analysis

Aortic tissues and RAECs were lysed in radioimmunoprecipitation assay buffer containing protease inhibitor. Protein concentrations were determined using a BCA Protein Assay Kit (Thermo Fisher Scientific, Waltham, MA). Equal amounts of protein were electrophoresed in SDS-polyacrylamide gels and subsequently transferred to membranes (Millipore). The membranes were probed with primary antibodies (Sigma-Aldrich [anti-β-actin mouse monoclonal antibody, #A5316, 1:3000 dilution], Abcam [anti-p53 rabbit pAb, #ab131442, 1:1000 dilution; anti-p21 rabbit mAb, #ab109199, 1:500 dilution; anti-p16 ARC rabbit mAb, #ab51243, 1:1000 dilution], Abclonal [anti-SIRT-1 rabbit pAb, #A11267, 1:1000 dilution], and Cell Signaling Technology [anti-eNOS rabbit mAb, #32,027, 1:1000 dilution] at 4 °C overnight, followed by incubation with a secondary horseradish peroxidase-conjugated anti-rabbit (#A0208, 1:1000 dilution) or anti-mouse IgG (#A0216, 1:1000 dilution; Beyotime Institute of Biotechnology, Shanghai, China) for 2 h at room temperature. Proteins were detected by chemiluminescence using enhanced chemiluminescence (ECL) reagent (Pierce, Rockford, IL). Quantity One (Bio-Rad, CA, USA) software was used to analyze the blots.

### Total antioxidant capacity (T-AOC) assay

Intracellular T-AOC was estimated using a commercial kit (Nanjing Jiancheng Bioengineering Institute, Nanjing, China) according to the manufacturer**’**s protocol. After the indicated treatment, RAECs were collected, centrifuged at 1000 rpm for 5 min, and resuspended in PBS. T-AOC was measured with a spectrophotometer (Thermo Fisher Scientific, Rockford, USA) at 520 nm.

### Measurement of the mitochondrial membrane potential (MMP)

The MMP was measured using the fluorescent lipophilic cationic dye JC‑1 (Invitrogen, USA) as described in a previous study [24]. Briefly, after the indicated treatments, RAECs were washed 3 times with PBS and incubated with 1 mg/L JC‑1 for 20 min at 37 °C. The cells were then washed with PBS and observed under a fluorescence microscope (Olympus). The cellular fluorescence intensity level selected from six random fields was analyzed using ImageJ software (NIH) at emission wavelengths of 590 nm (aggregates) and 535 nm (monomers). The ΔΨm of RAECs was calculated as the ratio of JC-1 aggregates to monomers (red/green).

### Transfection

RAECs were transfected with si-SIRT-1 or siRNA-negative control (siRNA-NC) using Lipofectamine™ RNAiMAX (Invitrogen) for 24 h according to the manufacturer’s protocol. Cells were inoculated into six-well plates and cultured until reaching 70–80% confluence. Meanwhile, 9 µL of Lipofectamine RNAiMAX Reagent was diluted in 150 µL of Opti-MEM, and 3 µL of SIRT-1 siRNA was diluted in 150 µL of Opti-MEM. Then, the same volume of the above solution was mixed for 5 min at room temperature. Finally, the siRNA-lipid complexes were added and incubated with the cells at 37 °C for 24 h. Transfection efficiency was determined by western blotting.

### Senescence-associated β-galactosidase staining

Senescence was detected with a senescence-associated-β-galactosidase (SA-β-Gal) staining kit (Cell Signaling Technology, Danvers, MA) following the manufacturer’s protocol as described in our previous study [14]. Images were obtained using an electron microscope (Olympus, Tokyo, Japan). The proportion of cells positive for SA-β-Gal staining is shown as the percentage of the total number of cells in each dish.

### Statistical analysis

Normally distributed data are expressed as the mean ± S.D. One-way analysis of variance (ANOVA) was used for comparisons in three or more groups, and the pairwise comparison was implemented with Tukey’s post hoc test. All statistical analyses were performed using SPSS 24.0 software. Statistical significance was set at *p* < 0.05.

## Results

### Basic biochemical characteristics of APS-treated old rats

To determine whether APS can improve oxidative stress and attenuate aortic endothelial senescence in old rats, 20-month-old rats were separately administered normal saline, low doses of APS (APS-L, 200 mg/kg), moderate doses of APS (APS-M, 400 mg/kg) or high doses of APS (APS-H, 800 mg/kg) by gavage every day for 3 months. The weights of the rats in the four groups were not significantly different before and after treatment (*p* > 0.05), as shown in Table 1. Moreover, ALT, Cr, glucose and LDL-C levels were not significantly different in the four groups after 3 months of treatment (Table 1).

**Table 1:** Comparison of weight and basic serum biochemical indicators in APS-treated old rats

	Con	APS-L	APS-M	APS-H
Weight (g) before treatment	750.50±125.56	749.00±121.04	741.63±164.44	742.63±103.32
Weight (g) after 3 m of treatment	733.33±144.08	732.33±109.35	726.50±117.28	629.33±17.39
ALT (U/L)	46.94±13.85	46.10±19.17	51.79±10.90	56.38±15.58
Cr (µmol/L)	110.47±37.69	105.89±43.47	88.13±28.13	94.05±25.34
Glu (mmol/L)	6.03±1.56	5.59±1.71	5.79±0.78	5.77 ±1.11
LDL-C (mmol/L)	4.12±1.14	3.75±1.25	2.80±0.86	3.11±0.68

Con: control group; APS-L: low-dose APS group; APS-M: moderate-dose APS group; APS-H: high-dose APS group; ALT: alanine aminotransferase; Cr: creatinine; Glu: blood glucose; LDL-C: low-density lipoprotein cholesterol

### APS improve old rat aortic morphology and have an anti-aging effect on old rat aortic endothelium

**Fig. 1:**
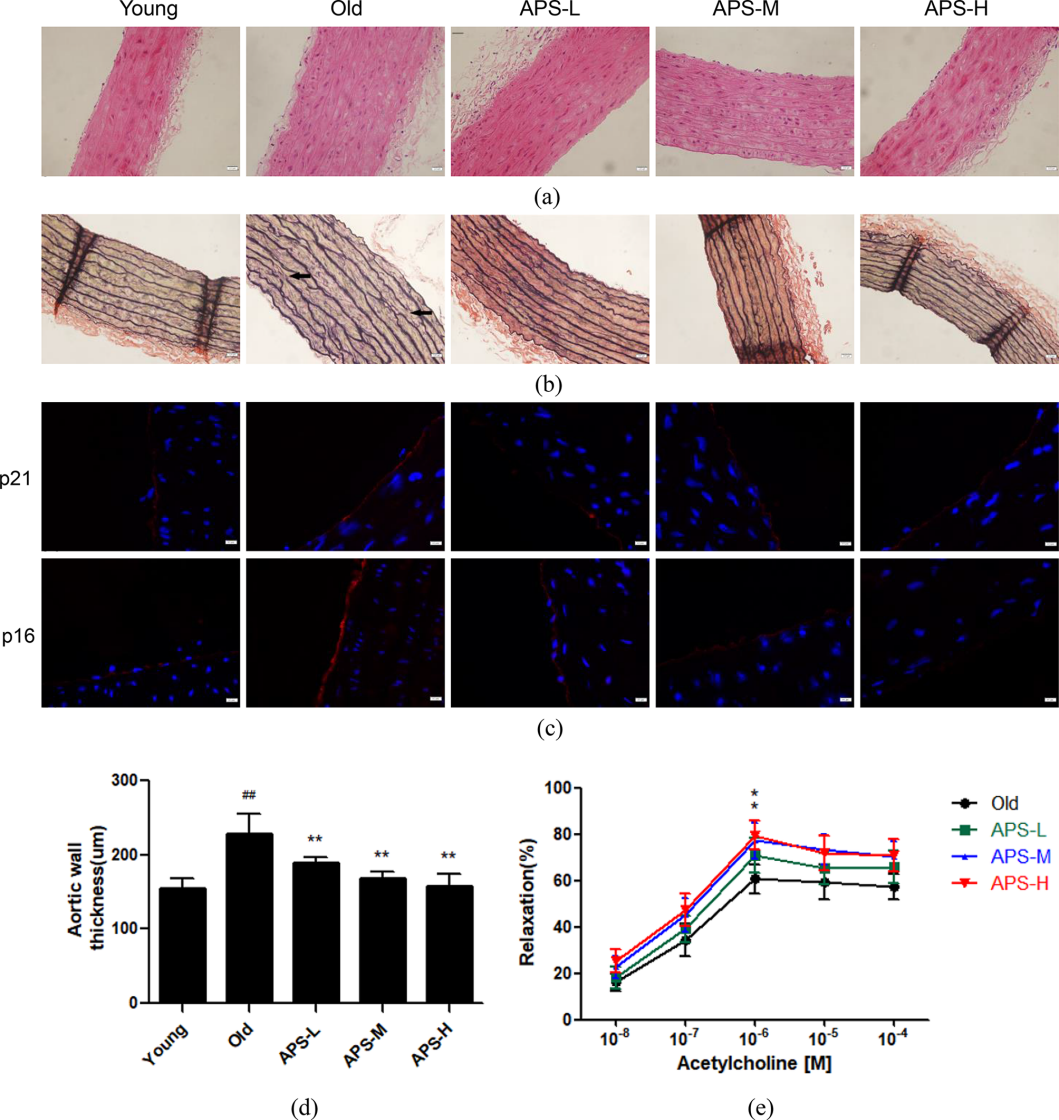
Effect of APS on aortic morphological changes in old rats. **(a)** Representative images of H&E staining and **(b)** VVG staining (black arrows show elastic fibers lysis). Magnification: 200×. Scale bar = 100 μm. **(c)** p21 and p16 expression in rat aortic endothelium was evaluated by immunofluorescence staining. Magnification: 400×. Scale bar = 40 μm. **(d)** Summary data of aortic wall thickness. Values are the means ± S.D. (*n* = 6). **(e)** Concentration-response curve of the vasodilator activity in old rats treated with different doses of APS. (*n* = 6). * *p* < 0.05, ** *p* < 0.01 vs. old group; ## *p* < 0.01 vs. young group. Young: young rat group; Old: old rat group; APS-L: low-dose APS group; APS-M: moderate-dose APS group; APS-H: high-dose APS group

H&E staining of aortic tissue slices showed that aortic wall thickness was increased in old rats (20-month-old rats) compared with that in young rats (2-month-old rats), and low-dose, moderate-dose and high-dose of APS all lessened senescent aortic wall thickness (*p* < 0.01, Fig. 1a and d). VVG staining, one of the most common stains to visualize vascular wall elastic tissue, showed that elastic fibers underwent lysis and exhibited a disorganized arrangement in the old rat group, and obvious improvement in elastic fiber integrity was visible in all APS-treated groups (Fig. 1b). In addition, we detected classic aging-related proteins p21 and p16 expression in the aortic endothelium using immunofluorescence staining (Fig. 1c). APS obviously weakened the fluorescence intensity of both p21 and p16 in old rats, which indicates that APS have an anti-aging effect on old rat aortic endothelium. Importantly, treatment with moderate and high doses of APS increased vasodilation at a concentration of 10^− 6^ mol/L acetylcholine (*p* < 0.05, Fig. 1e), suggesting APS may improve vascular endothelial function in old rats.

### APS reduce oxidative stress and increase SIRT-1 levels in old rats

**Fig. 2:**
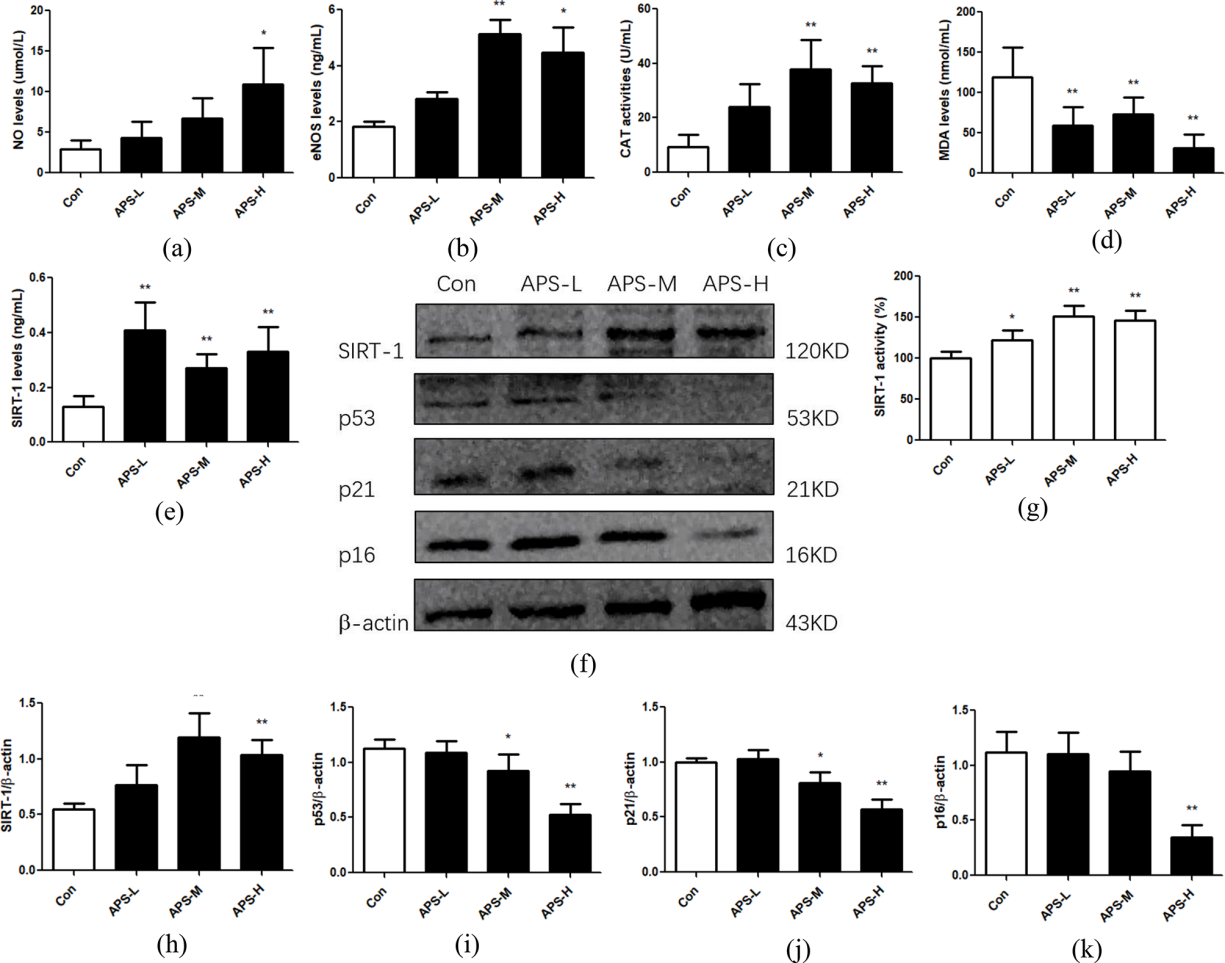
Effect of APS on serum NO and eNOS levels, oxidative stress indicators and senescent aortic tissue in old rats. **(a**-**e)** NO and eNOS concentrations, CAT activity, and MDA and SIRT-1 levels in the serum of rats. Values are the means ± S.D. (*n* = 6). **(f)** Western blotting bands showing expression levels of aging-related proteins, including SIRT-1, p53, p21 and p16, in old rat aortic tissues treated with low to high doses of APS. **(g)** SIRT-1 activity in aortic tissue homogenate from old rats treated with low to high doses of APS. The results are presented as a percentage of control group (taken as 100%). **(h**-**k)** Relative intensities of SIRT-1, p53, p21 and p16 proteins are shown. Values are the means ± S.D. (*n* = 6). * *p* < 0.05, ** *p* < 0.01 vs. control group. Con: old rat group as control group

NO levels were increased in the APS-H group, and eNOS levels were elevated in both the APS-M and APS-H group (Fig. 2a and b). The activity of the antioxidant enzyme CAT was increased in the APS-M and APS-H groups, whereas MDA, a metabolite of lipid peroxidation, was decreased in all the APS dose groups (Fig. 2c and d), which indicates that APS reduce oxidative stress in old rats.

Furthermore, the serum levels of the negatively related vascular aging indicator SIRT-1 were obviously increased in all the APS-treated groups (Fig. 2e), and both of the SIRT-1 protein levels and SIRT-1 activity increased in old rats treated with the moderate and high doses of APS for 3 months (*p* < 0.01, Fig. 2f and h). The expression of p53 and p21 proteins was decreased in the APS-M and APS-H groups, and p16 protein levels were lowered in the APS-H group (Fig. 2f, i and k).

### APS restore H_2_O_2_-induced impairment of mitochondrial function and total antioxidant capacity in RAECs

**Fig. 3:**
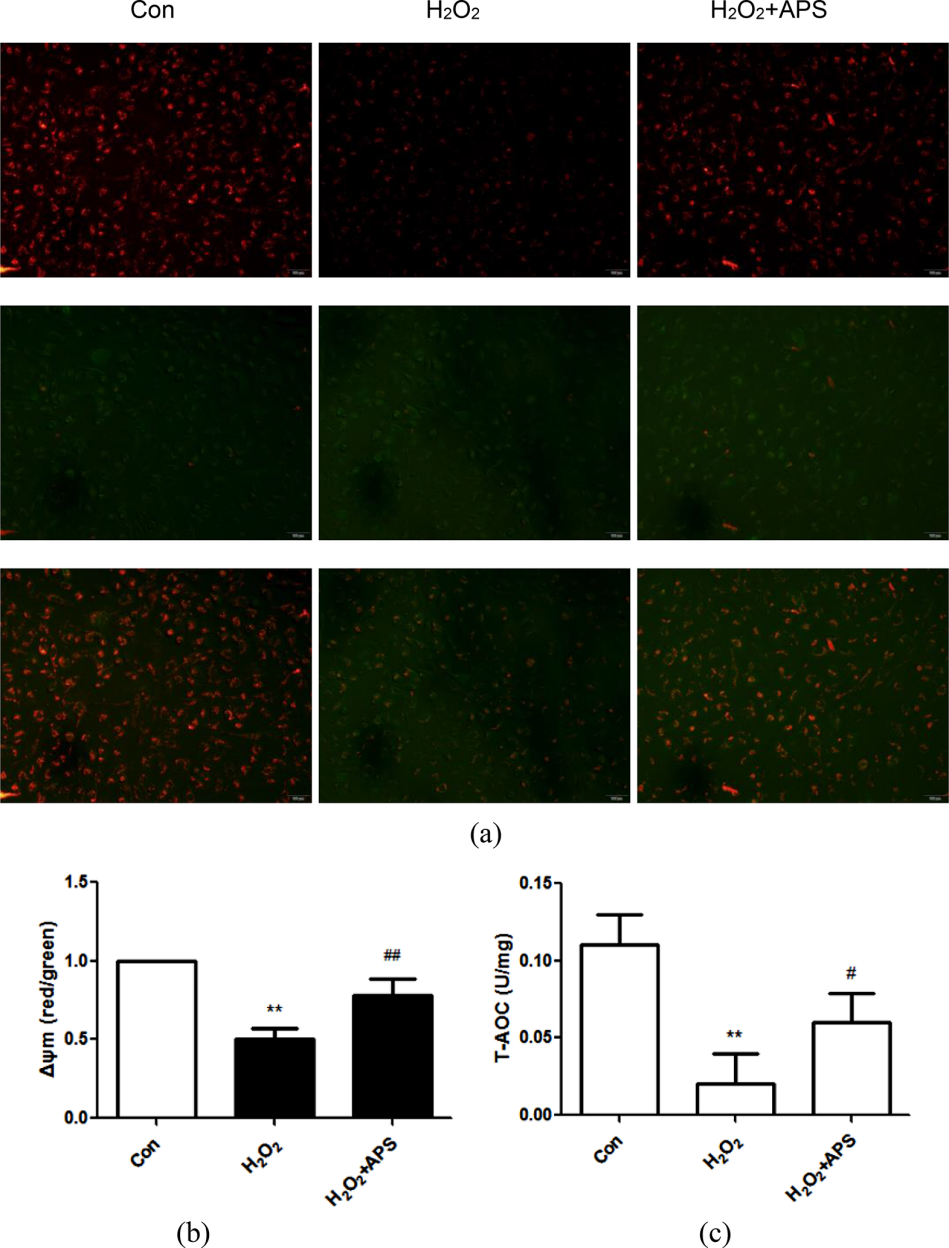
Effect of APS on the H_2_O_2_-induced mitochondrial membrane potential and total antioxidant capacity impairment in RAECs. **(a)** The mitochondrial membrane potential (MMP, Δψm) was observed using JC-1 staining. The red/green fluorescence intensity ratio was used to quantify the MMP. Magnification: 200×. Scale bar = 100 μm. **(b)** Summary ΔψM data are shown. **(c)** Intracellular total antioxidant capacity was determined with a T-AOC assay. Values are the means ± S.D. (*n* = 6). ** *p* < 0.01 vs. control (Con) group; # *p* < 0.05, ## *p* < 0.01 vs. H_2_O_2_ group

To verify the effect of APS on oxidative stress and senescence in RAECs, we isolated and cultured primary RAECs and tested whether APS can prevent oxidative stress-induced mitochondrial membrane depolarization and improve total antioxidant capacity (T-AOC) in RAECs. JC-1 staining was used to assess the MMP, which was calculated as the ratio of aggregates (red fluorescence) to monomers (green fluorescence) [24]. As shown in Fig. 3a and b, H_2_O_2_ lowered the red-to-green fluorescence intensity ratio, which was elevated by the addition of APS to RAECs. Moreover, APS obviously increased H_2_O_2_-induced T-AOC loss (Fig. 3c). These results indicate that APS restore H_2_O_2_-induced mitochondrial function impairment and improve T-AOC in RAECs.

### APS attenuate cellular senescence in RAECs

**Fig. 4:**
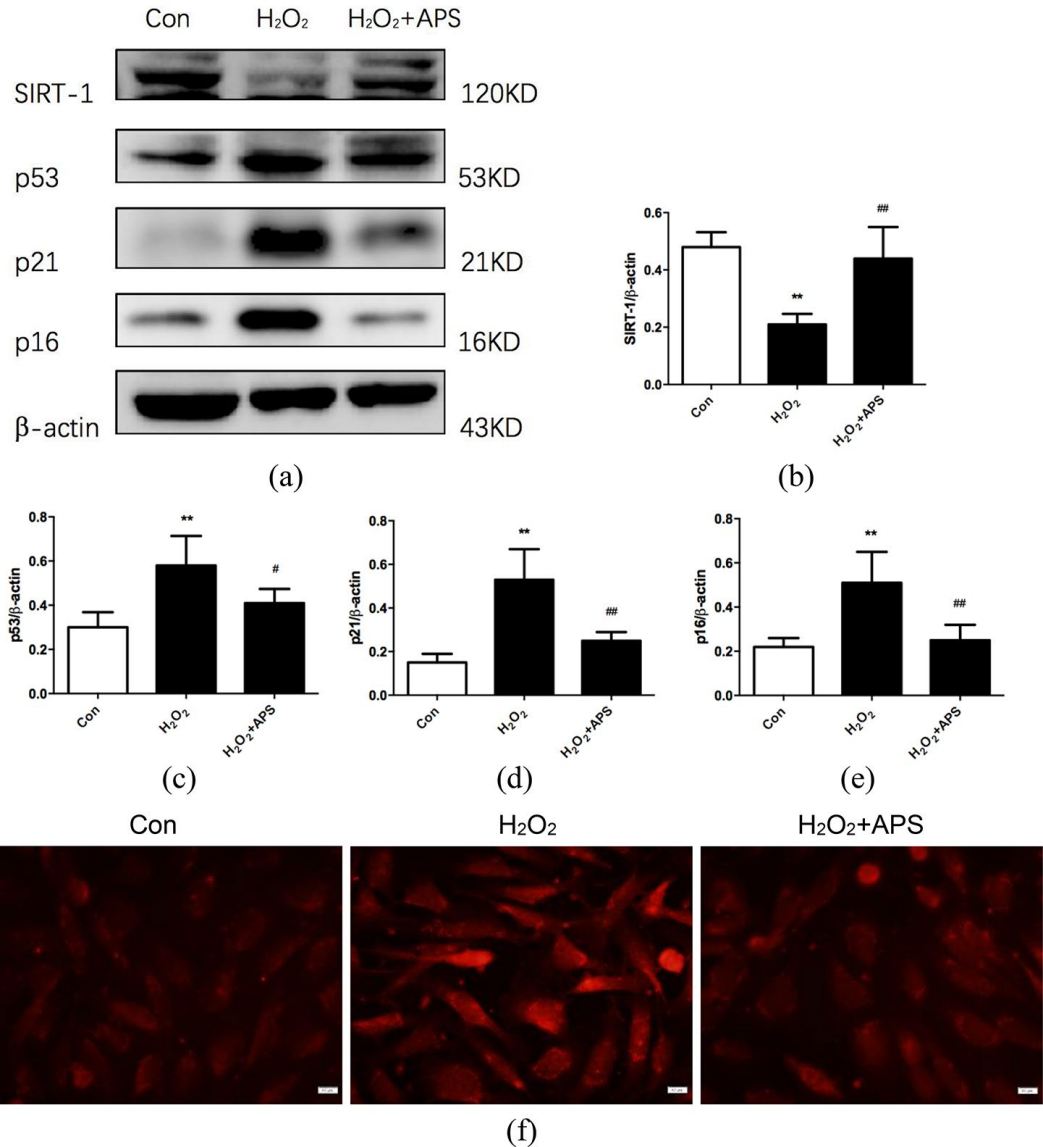
Effect of APS on H_2_O_2_-induced RAEC senescence. **(a)** Western blotting bands showing SIRT-1, p53, p21 and p16 expression in RAECs treated with H_2_O_2_ and APS. **(b**-**e)** Relative intensities of SIRT-1, p53, p21 and p16 in RAECs treated with H_2_O_2_ and APS. Values are the means ± S.D. (*n* = 6). **(f)** p16 expression in RAECs was evaluated by immunofluorescence staining. Magnification: 400×. Scale bar = 40 μm. ** *p* < 0.01 vs. control group; # *p* < 0.05, ## *p* < 0.01 vs. H_2_O_2_ group

**Fig. 5:**
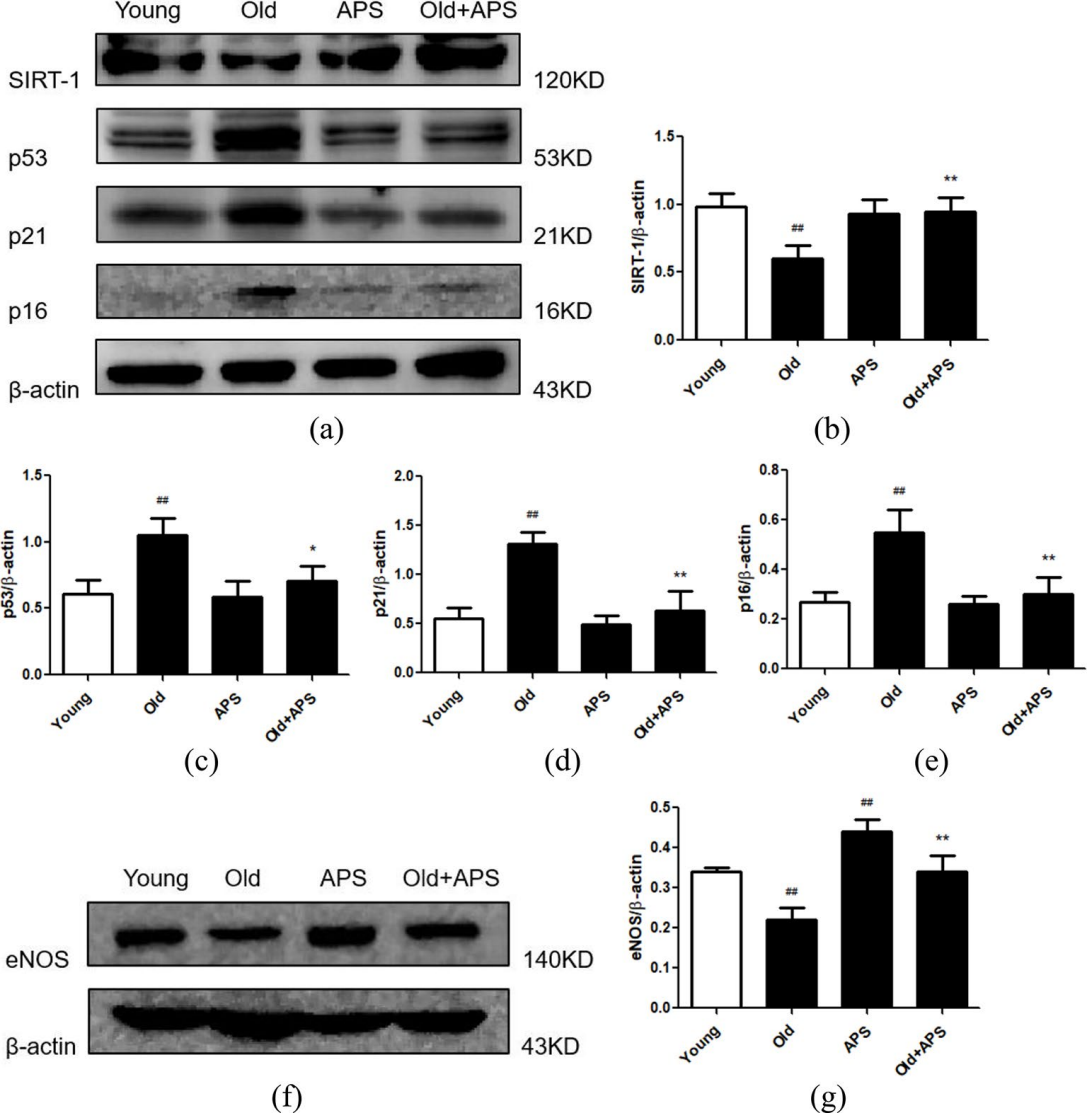
Effect of APS on primary RAEC senescence. **(a)** Western blotting bands showing SIRT-1, p53, p21 and p16 expression in RAECs isolated from young and old rats treated with APS. **(b**-**e)** Relative intensities of SIRT-1, p53, p21 and p16 in young and senescent RAECs treated with APS. (**f)** Western blotting bands showing eNOS expression in each group. **(g)** Relative intensities of eNOS protein bands are shown. Values are the means ± S.D. (*n* = 6). * *p* < 0.05, ** *p* < 0.01 vs. old group; ## *p* < 0.01 vs. young group. Young: RAECs isolated from 2-month-old rat group; Old: RAECs isolated from 20-month-old rat group; APS: the RAECs isolated from 2-month-old rats treated with APS group; Old + APS: the RAECs isolated from 20-month-old rats treated with APS group

We successfully established a senescent cell model using 100 µmmol/L H_2_O_2_-treated RAECs for 2 h and then cultured them in a complete medium for 48 h. Subsequently, we treated RAECs with 200 µg/mL APS for 24 h. The results showed that there was a significant reduction in SIRT-1 and an increase in p53/p21 and p16 protein expression in RAECs treated with H_2_O_2_, whereas APS effectively reversed these effects (Fig. 4). Therefore, we speculated that APS might enhance SIRT-1 expression and thus affect its downstream p53/p21 signaling pathways in RAECs. Moreover, we tested the above results in primary RAECs isolated from 20-month-old rats as another senescent cell model, and we confirm that APS have an anti-aging effect on senescent RAECs (Fig. 5a and e). Furthermore, the eNOS levels declined in aortic endothelial cells from old rats, whereas APS enhanced eNOS expression in senescent RAECs (Fig. 5f and g).

### Knockdown of SIRT-1 diminishes T-AOC and the anti-aging effect of APS in RAECs

**Fig. 6:**
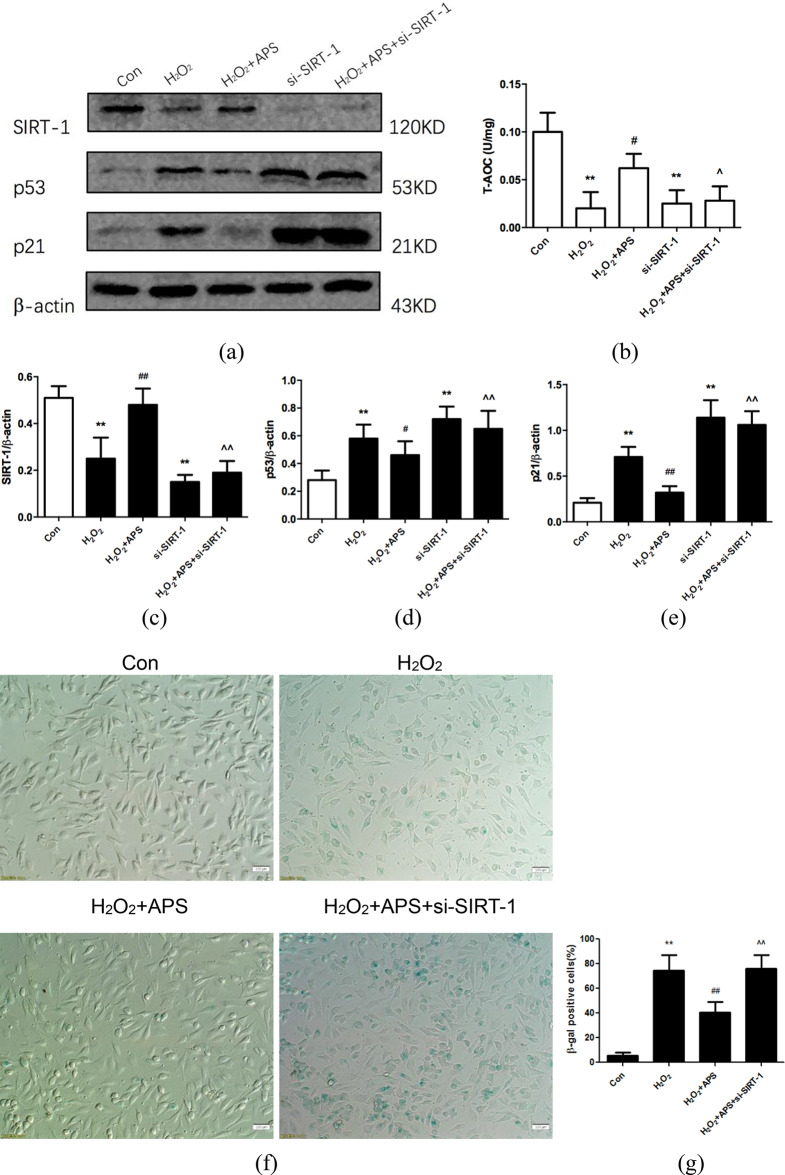
si-SIRT-1 reduces the APS-induced anti-aging effect in RAECs. **(a)** Western blotting bands showing SIRT-1, p53 and p21 expression. RAECs were pretransfected with siRNA-NC and then treated with normal medium (Con), 100 µmmol/L H_2_O_2_ (H_2_O_2_), or 200 µg/mL APS with H_2_O_2_ (H_2_O_2_ + APS) or were transfected with si-SIRT-1 (si-SIRT-1) and then treated with H_2_O_2_ and APS (HG + APS + si-SIRT-1). **(b)** Quantification of T-AOC in each group. **(c**-**e)** Relative intensities of SIRT-1, p53 and p21 protein bands are shown. **(f)** Representative images of SA-β-Gal-stained cells in each group. Magnification: 200×. Scale bar = 100 μm. **(g)** β-Gal-positive cells were quantified in each group. Values are the means ± S.D. (*n* = 6). ** *p* < 0.01 vs. control group; #*p* < 0.05, ## *p* < 0.01 vs. H_2_O_2_ group; ^*p* < 0.05, ^^*p* < 0.01 vs. H_2_O_2_ + APS group

To further confirm whether SIRT-1 is involved in the antioxidative and anti-aging effects of APS in RAECs, we knocked down SIRT-1 using target-specific RNA interference. The SIRT-1 protein level was significantly decreased by si-SIRT-1, as determined by western blotting (Fig. 6a). Next, we tested cellular T-AOC and found that knockdown of SIRT-1 diminished the effect of APS on H_2_O_2_-induced T-AOC loss (Fig. 6b). Subsequently, western blot analysis showed that knockdown of SIRT-1 increased the expression of the downstream proteins p53 and p21 and abated the protective effect of APS on H_2_O_2_-induced senescence (Fig. 6a, c and e). Furthermore, we used SA-β-gal staining to identify the presence of senescent cells [25] and confirm that APS decrease the percentage of SA-β-Gal-positive cells increased by H_2_O_2_, whereas knockdown of SIRT-1 diminishes the anti-aging effect of APS on RAECs treated with H_2_O_2_ (Fig. 6f and g).

## Discussion

**Fig. 7:**
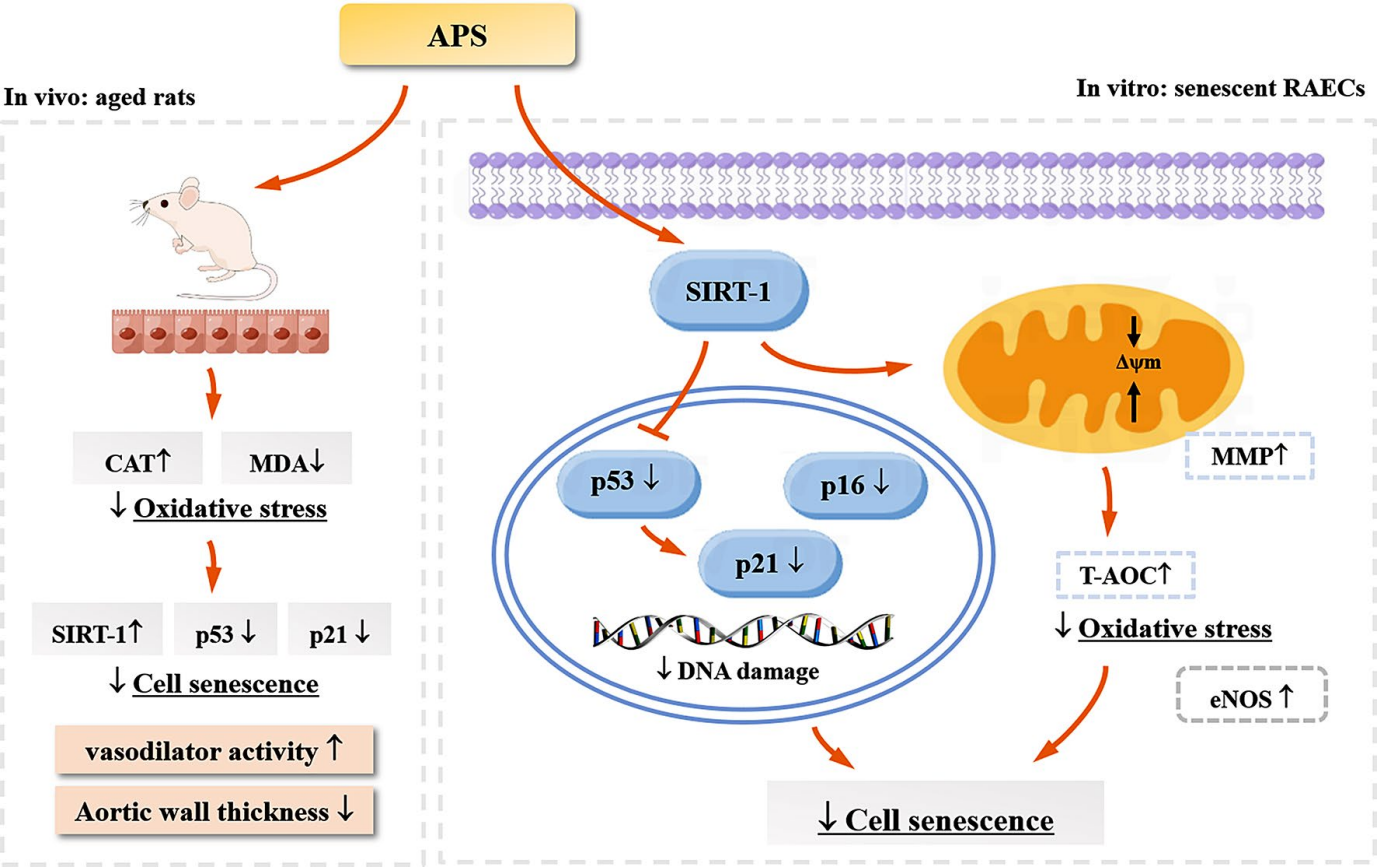
Schematic representation showing that APS may attenuate rat aortic endothelial senescence through the SIRT-1/p53 signaling pathway

Aging is an independent risk factor for atherosclerosis and impairs arterial function through decreased NO bioavailability and oxidative stress [26]. APS have been demonstrated to have antioxidative and anti-aging activities in mitochondria and BMSCs [11, 12]. Therefore, clarifying the effect of APS on artery endothelial senescence and finding the molecular targets involved may have potential for improving endothelial function and delaying the progression of aging-associated cardiovascular disease. In this study, we investigated the APS-induced anti-aging effect on natural aging rat aortic endothelium and primary RAECs, and found that APS may reduce rat aortic endothelial oxidative stress and senescence through the SIRT-1 signaling pathway (Fig. 7).

Oxidative stress, a major mechanism of aging, can result in vascular endothelial dysfunction, presenting with impairment of endothelium-dependent dilation (EDD), which can be evaluated by the reduction in NO bioavailability [27]. Endothelial NOS (eNOS) produced by the vascular endothelium regulates the biosynthesis of NO, which affects the cardiovascular system [28]. Our study found that APS increased vasodilation, serum NO and eNOS levels in old rats, and also increased eNOS protein expression in senescent RAECs. Furthermore, we assessed the antioxidant activity of APS by testing the activity of the antioxidant enzyme CAT and the levels of the metabolite of lipid peroxidation MDA [8]. Our data showed that APS increased CAT activity and decreased the MDA levels, which indicate that APS reduce oxidative stress in old rats. In vitro, we determined T-AOC and the MMP in H_2_O_2_-induced senescent RAECs. We found that H_2_O_2_ lowered T-AOC and that APS improved H_2_O_2_-induced T-AOC loss in RAECs. Oxidative stress has been demonstrated to increase the mitochondrial membrane depolarization and thus lead to cell death [29], which can be determined by JC-1 fluorescence staining [21]. Our results showed that H_2_O_2_ reduced the ΔΨm, which was prevented by APS supplementation, indicating that APS ameliorate oxidative stress-induced mitochondrial function impairment in RAECs.

Cell senescence is marked by increased expression of several genes, including the cyclin-dependent kinase inhibitors p21 and p16 [30, 31]. Endothelial dysfunction in senescent arteries is dependent on p53 pathways and maintained by upregulation of p21-mediated cell growth arrest [32]. In this study, 400 mg/kg/d and 800 mg/kg/d APS given by gavage for 3 months decreased p53 and p21 protein expression, and high doses of APS lowered p16 protein levels in old rat aortic tissue. Considering the mixed effects of other cells in aortic tissue, we further demonstrated that APS decreased both p21 and p16 protein immunofluorescence intensities in senescent aortic endothelium. H_2_O_2_ is widely applied in many studies to examine the effects of oxidative stress-induced senescence [33]. In the present study, we established two kinds of senescent cell models including H_2_O_2_-induced senescent RAECs isolated from 2-month-old rats and the RAECs isolated from natural aging rats. In both of the senescent cell models, we found that APS lowered aging-related protein expression. Therefore, our results demonstrate that APS attenuate rat aortic endothelial senescence in vitro and in vivo.

SIRT-1, which is highly expressed in endothelial cells, plays an essential role in regulation of vascular endothelial function through deacetylation of eNOS, improving the bioavailability of NO [34, 35]. Notably, studies have demonstrated that SIRT-1 expression significantly declines with age, and low expression of SIRT-1 promotes vascular aging in endothelial cells [20, 36], indicating that SIRT-1 is a central factor involved in vascular endothelial aging [19]. However, several recent studies described that SIRT-1 overexpression is associated with augmented levels of oxidative stress, and resultant hyperproliferation of vascular smooth muscle cells [37, 38], which suggest that SIRT-1 may exert both protective and deleterious effects on the cardiovascular system. Alcendor et al. found that SIRT-1 exhibited protective and harmful effects in cardiac-specific transgenic mice depending on the degree of overexpression of SIRT-1, mild to moderate expression of SIRT-1 reduced oxidative stress and retarded aging of the heart, whereas a high dose of SIRT-1 increased oxidative stress [39]. In the present study, our results indicate that the enhancement of SIRT-1 expression by APS reduce oxidative stress in RAECs. APS, a bioactive component of *Astragalus*, have been reported to ameliorate stress in muscle mitochondria and retinal pigment epithelial cells via a SIRT-1-related pathway [21, 40]. Whether SIRT-1 is involved in APS-mediated regulation of aortic endothelial aging is unclear. Our results found that APS treatment increased serum SIRT-1, eNOS and NO levels in old rats and enhanced SIRT-1, p53 and p21 protein expression in old rat aortic tissue. Moreover, we demonstrated APS enhanced SIRT-1 activity in aging rat aortae and increased SIRT-1 protein expression in senescent RAECs. These data suggest that APS may reduce aortic endothelial senescence through SIRT-1-related signaling pathways.

The anti-aging activity of SIRT-1 is considered to be predominately linked to SIRT-1-induced deacetylation of p53. Cellular senescence is usually characterized by elevated levels of p53 during oxidative stress, and p21 is an important downstream target of p53 that participates in cell cycle arrest, which marks cellular aging [41]. Lamichane et al. found that overexpression of SIRT-1 prevented stress-induced endothelial progenitor cell senescence by inhibiting the p53/p21 pathway [42], and the same protective mechanism was found in adipose tissue-derived mesenchymal stem cell senescence [43]. Consistent with previous studies, our results showed that knockdown of SIRT-1 increased downstream p53 and p21 protein expression, and APS decreased RAEC senescence induced by H_2_O_2_, whereas downregulation of SIRT-1 diminished the protective effect of APS on H_2_O_2_-induced T-AOC loss and cellular aging, indicating that the SIRT-1/p53/p21 signaling pathway may be involved in the anti-aging effect of APS on rat aortic endothelium.

The limitations of the present study include that the ingredients of APS may be metabolized into a new structure in vivo, and the circulation metabolites of APS and their effect on vascular endothelial cell senescence should be further verified. Furthermore, there were no positive and negative control drug groups which can make the results more rigorous in this study.

## Conclusions

In summary, our study suggests that APS attenuate rat aortic endothelial oxidative stress and senescence. The SIRT-1/p53 signaling pathway may be involved in its underlying mechanism. APS and SIRT-1-targeted drugs have great potential for the treatment of aging-associated atherosclerosis and cardiovascular disease.

## Data Availability

The data and materials supporting this study are available with the corresponding author upon request.

## Abbreviations

APS: Astragalus polysaccharides
RAECs: rat aortic endothelial cells
H&E: hematoxylin and eosin
VVG: Verhoeff Van Gieson
MMP: mitochondrial membrane potential
SA-β-Gal: senescence-associated-β-galactosidase
SIRT-1: Sirtuin 1; H_2_O_2_: hydrogen peroxide
T-AOC: total antioxidant capacity
NO: nitric oxide
eNOS: endothelial nitric oxide synthase
ROS: reactive oxygen species
BMSCs: bone marrow mesenchymal stem cells
AMPK: AMP-activated protein kinase
SPF: specific pathogen free
ALT: Alanine aminotransferase
Glu: blood glucose
LDL-C: low-density lipoprotein cholesterol
CAT: catalase
MDA: malondialdehyde
ELISA: enzyme-linked immunosorbent assay
DAPI: 4’,6-diamidino-2-phenylindole
EDD: endothelium-dependent dilation
